# Untargeted Metabolomics Reveals Alterations of Rhythmic Pulmonary Metabolism in IPF

**DOI:** 10.3390/metabo13101069

**Published:** 2023-10-10

**Authors:** Wei Sun, Jiuqiang Ren, Zixian Jia, Puyang Liang, Shengxi Li, Meiyue Song, Yinghao Cao, Haoran Chen, Qiang Luo, Lifeng Yang, Jing Wang, Chen Wang, Lin Wang

**Affiliations:** 1 Department of Respiratory and Critical Care, The Second Hospital of Jilin University, 218 Ziqiang Street, Changchun 130012, China; 2 State Key Laboratory of Common Mechanism Research for Major Disease, Institute of Basic Medical Sciences, Chinese Academy of Medical Sciences and Peking Union Medical College, Beijing 100730, Chinas2022005020@pumc.edu.cn (H.C.); 3 Shanghai Institute of Nutrition and Health, University of Chinese Academy of Sciences, Shanghai 200031, China; 4 State Key Laboratory of Respiratory Health and Multimorbidity, Institute of Basic Medical Sciences Chinese Academy of Medical Sciences, School of Basic Medicine Peking Union Medical College, Beijing 100005, Chinawangjing@ibms.pumc.edu.cn (J.W.); 5 Department of Cardiology, The Second Hospital of Jilin University, 218 Ziqiang Street, Changchun 130012, China; 6 Department of Pathophysiology, Institute of Basic Medical Sciences, Chinese Academy of Medical Sciences and Peking Union Medical College, Beijing 100730, China

**Keywords:** idiopathic pulmonary fibrosis, untargeted metabolomics analysis, rhythm

## Abstract

Idiopathic pulmonary fibrosis (IPF) is a chronic and progressive condition characterized by the impairment of alveolar epithelial cells. Despite continued research efforts, the effective therapeutic medication is still absent due to an incomplete understanding of the underlying etiology. It has been shown that rhythmic alterations are of significant importance in the pathophysiology of IPF. However, a comprehensive understanding of how metabolite level changes with circadian rhythms in individuals with IPF is lacking. Here, we constructed an extensive metabolite database by utilizing an unbiased reference system culturing with ^13^C or ^15^N labeled nutrients. Using LC-MS analysis via ESI and APCI ion sources, 1300 potential water-soluble metabolites were characterized and applied to evaluate the metabolic changes with rhythm in the lung from both wild-type mice and mice with IPF. The metabolites, such as glycerophospholipids and amino acids, in WT mice exhibited notable rhythmic oscillations. The concentrations of phospholipids reached the highest during the fast state, while those of amino acids reached their peak during fed state. Similar diurnal variations in the metabolite rhythm of amino acids and phospholipids were also observed in IPF mice. Although the rhythmic oscillation of metabolites in the urea cycle remained unchanged, there was a significant up-regulation in their levels in the lungs of IPF mice. ^15^N-ammonia in vivo isotope tracing further showed an increase in urea cycle activity in the lungs of mice with IPF, which may compensate for the reduced efficiency of the hepatic urea cycle. In sum, our metabolomics database and method provide evidence of the periodic changes in lung metabolites, thereby offering valuable insights to advance our understanding of metabolic reprogramming in the context of IPF.

## 1. Introduction

Metabolism is a complex network of biochemical reactions. One of its primary roles is to provide energy to sustain life. Humans, like all mammalian species, coordinate and reprogram their metabolic reactions with circadian rhythms, which are composed of multiple tissue-specific oscillators [1,2,3]. The central clock residing in the suprachiasmatic nucleus (SCN) of the brain is predominantly regulated by the light-dark cycle. The feeding-fasting cycle, however, is believed to affect the clocks of peripheral tissues such as the diaphragm, liver, stomach, intestine, and kidney [4,5]. Circadian rhythm control occurs at all levels, ranging from the transcriptional regulation of gene expression to the posttranslational modification of existing proteins. Recently, it has been discovered that circadian rhythms also play a significant role in regulating metabolite levels in both circulation and tissues. For example, individuals who consume identical meals during breakfast, lunch, or dinner experience the lowest postprandial blood glucose levels following breakfast, whereas the highest levels are observed after dinner [6]. Furthermore, it has been observed that in the case of healthy adults who undergo a continuous glucose infusion over a period of 24 h, there is a notable increase in blood glucose levels during the night, followed by a subsequent fall at dawn [7]. In addition, lipids metabolism also exhibits a circadian rhythm, with elevated levels of triacylglycerol in the bloodstream throughout the biological night and a greater postprandial response following a night-time meal compared to that during the day [8]. Disruption of such circadian rhythms causes metabolic disorders, including obesity [9,10], type 2 diabetes [11], and neurological disorders [12].

Unlike the brain and other peripheral organs, pulmonary metabolic rhythms are also controlled by the daily oscillation in airway caliber. Long-term pathological progression, hypercapnia, and inhaled allergen can cause dysrhythmia of the lung [13,14,15]. The clinical significance of rhythms and the molecular biology underlying this has long been recognized. Several molecular processes, including nicotinamide (NAD) oxidation [16,17], Nrf2 activity [18,19], glutathione reduction [19], and peroxiredoxin activity [16,20,21] are implicated in the dysregulation of pulmonary rhythm. Additionally, the exhaled volatile organic compounds (VOCs) exhibited proportional rhythmic variation over 24 h [22]. The metabolic signals and states can give feedback to the circadian rhythm with crucial input. For instance, cellular NAD^+^ levels influence mitochondrial oxidative metabolism [23] via the participation of sirtuins such as SIRT1, SIRT3, and SIRT6 [17,18,24], which in turn regulate the expression of oxidative enzymes, mitochondrial metabolism, and cellular respiration [25]. The intricate interaction between the rhythmic oscillation of pulmonary metabolite and the molecular clock highlights the significance of the relationship between metabolism and circadian regulation in maintaining pulmonary health.

Idiopathic pulmonary fibrosis (IPF) is a chronic interstitial lung disease characterized by diffuse alveolitis and structural abnormalities in the alveoli. It is recognized as a disease involving multiple complex factors, including macrophage activation, activation of the transforming growth factor (TGF-ɑ) pathway, fibroproliferative responses resulting from aberrant kinase activation, and reactivation of developmental pathways [26,27,28]. Despite extensive research on the IPF-developed mechanism, the mortality rate of IPF is still high, with a 5-year survival rate ranging from 30% to 50% and an average survival time of less than 3 years [29]. Recent studies have demonstrated that the hallmark features of pulmonary fibrosis (PF), such as extracellular matrix (ECM) remodeling, epithelial-mesenchymal transition (EMT), and fibroblast-to-myofibroblast differentiation, can be regulated by the transcription of clock-controlled genes (CCGs) [30]. REV-ERBα (NR1D1) is one of the core circadian clock factors that has been shown to prevent the progression of PF [31]. Previous studies have demonstrated that REV-ERBα exhibits peak expression levels during nocturnal periods in wild-type mice. Conversely, in the context of IPF, it appears to undergo downregulation, specifically during nocturnal intervals inside the lung tissue [32]. The administration of an Nrf2 activator (sulforaphane) or NR1D1 agonist (SR9009) gives the potential to mitigate the temporal protective response [19,32]. These findings highlight the significant role of the biological clock in regulating the pathological progression of IPF [33] and motivate us to completely comprehend the regulatory role of the molecular clock in IPF metabolism, which may present enormous therapeutic opportunities.

Herein, we construct a database using ^13^C and ^15^N isotope-labeled yeast in conjunction with advanced ion source detection techniques, such as electrospray ionization (ESI) and atmospheric pressure chemical ionization (APCI). Using our previously developed system Peak Annotation and Verification Engine (PAVE) [34], a database containing 1300 identified metabolites is established. Using a combination of metabolomics, RNAseq, and in vivo isotope tracing, we further investigate the rhythmic oscillation of metabolites in both wild-type and bleomycin-induced IPF mice. Several metabolic pathways related to lipid metabolism and amino acids have notable oscillations in both WT and IPF lung rhythm. An accumulation of urea cycle metabolites and a subtle elevation in the ^15^N labeling flux of the urea cycle was observed in the IPF lung. The potential disruption of the urea cycle rhythm in the lungs of IPF may offer valuable insights for the development of innovative treatment strategies, such as optimizing time-based therapy to target the urea cycle.

## 2. Methods

### 2.1. Animal Model

Mice studies followed protocols approved by the Institute of Basic Medical Sciences (IBMS)/Peking Union Medical College (PUMC) Animal Care and Use Committee. 8–10-week-old male C57BL/6J were group-housed on a light-dark cycle (9 am–9 pm) with free access to water and chow (Jiangsu-Xietong, XT101FZ-002, No. 11 Juyuan Road, Guli Industrial Park, Jiangning District, Nanjing, China). The temperature in the mouse feeding room was kept at 22 ± 1 °C with relative room humidity: 50–60%, air exchange: 8–20 h^−1^, air flow: 10–25 cm/min, noise: below 60 dB. To generate the IPF model, mice were injected intratracheally with 1.5–2 U/kg bleomycin. The rodents in the control group received the same volume of sterile Phosphate Buffered Saline (PBS). Six mice from the IPF group and four mice from the control group were sacrificed every four hours for untargeted metabolomics analysis. RNA sequencing (RNA-seq) was concurrently performed on the same lung tissues.

### 2.2. Yeast Culture

*Saccharomyces cerevisiae* strain CENPK was grown at 30 °C in Synthetic Dextrose (Difco™ DF0919) medium with 0.5% [U-^15^N_2_] ammonium sulfate and 2% [U-^13^C_6_] glucose. Labeling was carried out for >10 generations. Cells were harvested in the exponential phase (OD_600_ of 0.8, 2 mL) by centrifuging at 3000 rpm for 15 min at 4 °C. Then, 1 mL 80%:20% methanol:water were added for extraction. The resulting mixture was transferred into an Eppendorf tube and spun down at 15,000× *g* for 40 min at 4 °C. The supernatant was taken for LC−MS analysis.

### 2.3. Detection of Lung Function in Mice

The mice were anesthetized with intraperitoneal injection of tribromoethanol. Subsequently, they were immobilized on the operating table, where the skin was incised and the trachea was carefully dissected and exposed. The tracheal catheter was inserted and securely fastened. Endotracheal intubation was connected to the pulmonary function instrument (SCIREQ-FlexiVent), and the pulmonary function includes functional residual capacity (FRC), inspiratory capacity (IC), resistance to inhalation (RI), dynamic lung compliance (Cydn) was recorded. All parameters were measured more than three times, and the results were averaged values.

### 2.4. RNA-Seq Analysis and Bioinformatics

Prior to library preparation, the integrity and purity of RNA were inspected. The sequencing libraries were constructed according to the manufacturer’s instructions using a TruSeq PE Cluster Kit v3-cBot-HS (Illumina) and then sequenced in 150-bp paired-end reads on an Illumina Hiseq platform (Novogene Co., Ltd., Beijing, China). Using HISAT2 with the default parameters [35], the purified reads of each sample were aligned to their respective reference genomes (mouse: GRCm38). Using the GenomicFeatures and GenomicAlignments packages in R 4.2.3 [36], the read counts of each gene were then computed. Using the DESeq2 package [37], the gene expression levels (Fragments per Kilobase Million; FPKM) and significance analyses for differentially expressed genes were obtained. The time clustering curve is drawn using the R (fMuzz) program.

### 2.5. Extraction of Metabolites from Tissue and Serum

Tissues were collected and promptly frozen in liquid nitrogen using a clamp. Then, the tissues were stored at −80 °C before extraction. Frozen tissues were ground using a Cyromill at cryogenic temperature. The ground tissue was then weighed and mixed with −20 °C 80%:20% methanol:water at a concentration of 1 mg/40 µL. Samples were briefly vortexed and centrifuged twice at 16,000× *g* for 30 min at 4 °C before the final supernatant was transferred to liquid chromatography-mass spectrometry (LC-MS) tubes for analysis. Blood samples were drawn from the tail vein and stored on ice. After 10 min of centrifugation at 4 °C and 4500 rpm, serum was collected in a 1.5 mL tube and stored at −80 °C for future analysis. For serum extraction, 2 µL of serum was combined with 80 µL of methanol-based extraction reagent (1:40). After centrifugation (30 min, 16,000× *g*, 4 °C.), the supernatant was transferred to an LC-MS vial for analysis.

### 2.6. LC-MS Analysis

To measure metabolites in serum and tissue samples, an orbitrap mass spectrometer (Orbitrap Exploris 480; Thermo Fisher Scientific) was coupled to a Vanquish UHPLC system (Thermo Fisher Scientific) with electrospray ionization (ESI) or atmospheric pressure chemical ionization (APCI) and a scan range of *m/z* from 70 to 1000, with a 120,000 resolution. A gradient of solvent A (95:5 water: acetonitrile containing 20 mM of ammonium acetate and 20 mM of ammonium hydroxide, pH 9.45) and solvent B (acetonitrile) was utilized for LC separation on an XBridge BEH Amide column (2.1150 mm, 2.5 m particle size; Waters). The rate of flow was 150 μL/min. The LC gradient was: 0 min, 90%B; 2 min, 90% B; 3 min, 75%; 7 min, 75% B; 8 min, 70% B, 9 min, 70% B; 10 min, 50% B; 12 min, 50% B; 13 min, 25% B; 14 min, 25% B; 16 min, 0% B, 21 min, 0% B;21 min, 90% B; and 25 min, 90% B. The injection volume was 10 μL, and the temperature of the autosampler was set to 4 °C. Metabolite peaks were picked with signal to noise (S/N) at S/N > 5.

### 2.7. ^15^N-Ammonia Injection

330 mmol/l of ^15^NH_4_Cl was a retro-orbital injection into the bloodstream after mice were anesthetized with isoflurane. For metabolic analysis, tail vein blood and lung tissue were collected at 0 min, 2 min, 10 min, and 30 min after cervical dislocation.

### 2.8. Statistical Analysis

The statistical significance was determined using either a two-tailed, unpaired Student’s *t*-test or a two-way analysis of variance (ANOVA) for group comparisons. A *p*-value of less than 0.05 was statistically significant. All statistical analysis was conducted using Prism version 8.0 (GraphPad, La Jolla, CA, USA).

## 3. Results

### 3.1. Establishment of the Metabolomics Database

**Figure 1:**
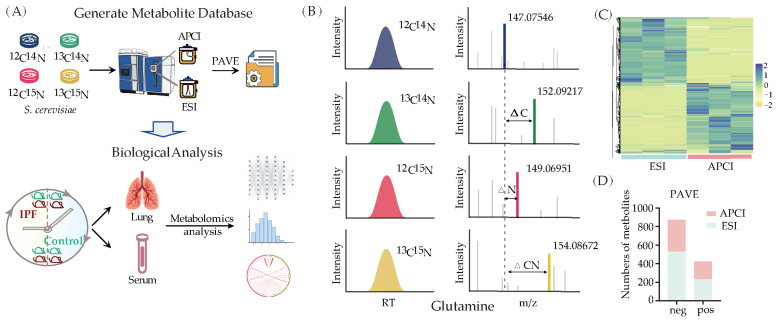
Establishment of a non-targeted metabolomics database. (**A**) Database establishment process and application. ^13^C, ^15^N-labeled yeast was used for ESI-LC-MS and APCI-LC-MS analysis. After PAVE screening, we obtained pseudo-metabolites that matched with standards or MS2. (**B**) Screening metabolites according to the intensity and mass shift among ^13^C and ^15^N labeled samples in the LC-MS. (**C**) Heat maps of metabolites measured using ESI and APCI ion source (*n* = 3). (**D**) Metabolites number detected using different ion sources.

Because of the rapid growth rate and simple culture conditions, *S. cerevisiae* was employed to generate a comprehensive metabolite database by culturing in four distinct isotopic conditions (unlabeled medium, ^15^N-labeled medium, ^13^C-labeled medium, and ^15^N^13^C-labeled medium) (Figure 1A). For LC-MS-based untargeted metabolic analysis, both ESI and APCI ion sources were used. We investigated how voltage and extraction solvents affected the detection results. Results show that using 80% methanol as an extraction solvent led to better detection with the ESI ion source, whereas using 100% methanol for extraction yielded superior results with the APCI ion source (Figure S1A). The mass shift across the four designated conditions (Figure 1B) was then inputted into our previously published formula prediction engine PAVE [34] for formula prediction. We obtained 1300 putative metabolite chemical compounds. Among these, in the negative ion mode, 347 metabolites were detected using the APCI ion source and 526 using the ESI ion source; in the positive mode, 192 metabolites were detected using APCI and 232 metabolites were detected using the ESI ion source (Figure 1C). Further evidence of the advantages of particular ion sources for the identification of metabolites was provided in the heat map (Figure 1D). To derive metabolite names, the putative metabolite chemical formulas were confirmed using exact mass, retention time match to authenticated standards, or tandem mass spectrometry (MS2) match to the Human Metabolome Database (HMDB) [38]. By employing this methodology, we have compiled a comprehensive database of detected metabolites that can be used to resolve our specific biological questions and broaden our knowledge of cellular metabolism.

### 3.2. Rhythmic Oscillation of Metabolic Changes in the Lung of Normal Mice

**Figure 2:**
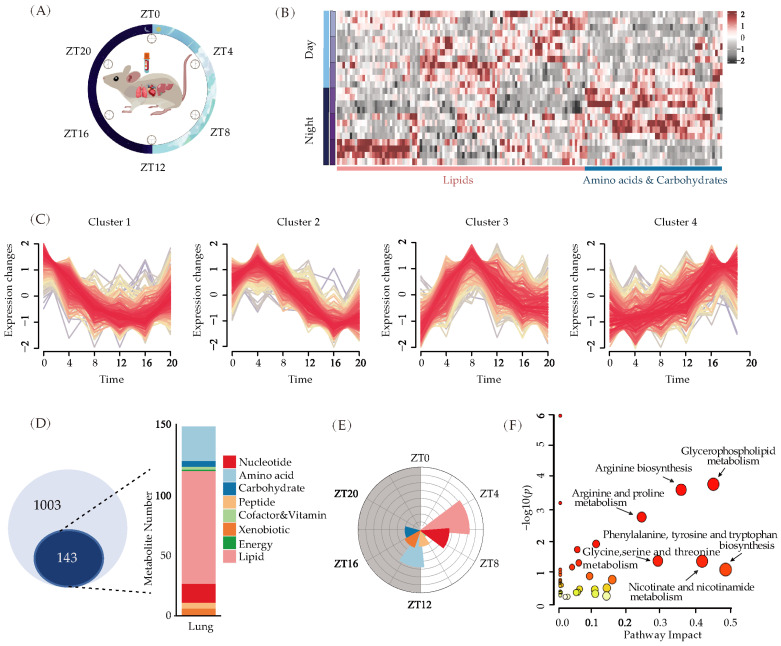
Rhythmic oscillation of metabolites in the lung under physiological state. (**A**) The time course for metabolic analysis. ZT0 was light on, and ZT12 was light off. Samples of blood, lung, and liver tissues were obtained at regular intervals of four hours for metabolomics analysis. (**B**) Heatmap of liver metabolites (*n* = 4). (**C**) Temporal clustering curve of metabolites in lung under physiological state (*n* = 6). (**D**) Metabolites measured in the lung of WT mice. A total of 1003 metabolites were detected, of which 143 exhibited rhythmic oscillations. Lipids had the highest frequency of rhythmic oscillations among the classified metabolites, with amino acids following after (*n* = 6). (**E**) The rose diagram of the peak time of metabolite classification. (**F**) The pathway enrichment about metabolites with rhythmic oscillations. Size and color of dot indicate significance of regulated (−log10(*p*)) metabolites.

There has been an increasing amount of research indicating that metabolic changes in the lungs play a crucial role in respiratory system diseases. To understand how the rhythmic oscillation of metabolites works in the respiratory system, we sampled tissues and blood every 4 h throughout the light/dark cycle (where ZT0 represents light-on and ZT12 represents light-off) and conducted untargeted metabolite profiling using LC-MS (Figure 2A). Since it is known that peripheral oscillators in the liver play fundamental roles in maintaining energy metabolism, we examined the rhythm of energy metabolites in both the lung and liver under physiological conditions to ensure the time course we chose works for the circadian clock. A total of 1271 metabolites were identified in the tissues (Figure S2A), and 759 of them were detected in both the liver and the lung. In the liver, it is evident that amino acids and carbohydrates displayed maximal levels during the night, whereas lipid substances displayed a continuous 24 h oscillation (Figure 2B). This is consistent with previous literature reports [8] demonstrating our entire experimental procedure.

Next, we analyzed the rhythm of respiratory metabolism. Temporal clustering analysis of 1003 measured metabolites revealed that certain metabolites peaked during the day, whereas others peaked at night, exhibiting rhythmic oscillations over time (Figure 2C). Specifically, 143 metabolites in the lung tissue have rhythmic oscillations, with lipid and amino acid metabolites exhibiting the most notable alterations (Figure 2D). Lipids, nucleic acids, and peptides attained their peak levels during the day, whereas amino acids and carbohydrates peaked at night (Figure 2E). These results indicate a relationship between the metabolic properties of these substances and their temporal regulation. For instance, it is known that lipid metabolism species exhibit daily cycles, with higher levels of nonesterified fatty acids (NEFA) in the circulation of rodents during their inactive phase [39,40], while during the awake phase, circulating amino acid levels are elevated, serving as substrates and stimuli for protein synthesis [41]. The metabolic pathways of glycerophospholipid, arginine, proline, phenylalanine, tyrosine, tryptophan, and glycine-serine-threonine are enriched in these rhythmic metabolites (Figure 2F). Together, under physiological conditions, these data indicate a circadian oscillation of a specific metabolite in the lungs.

### 3.3. Pulmonary Fibrosis Disorders Lung Rhythm

**Figure 3:**
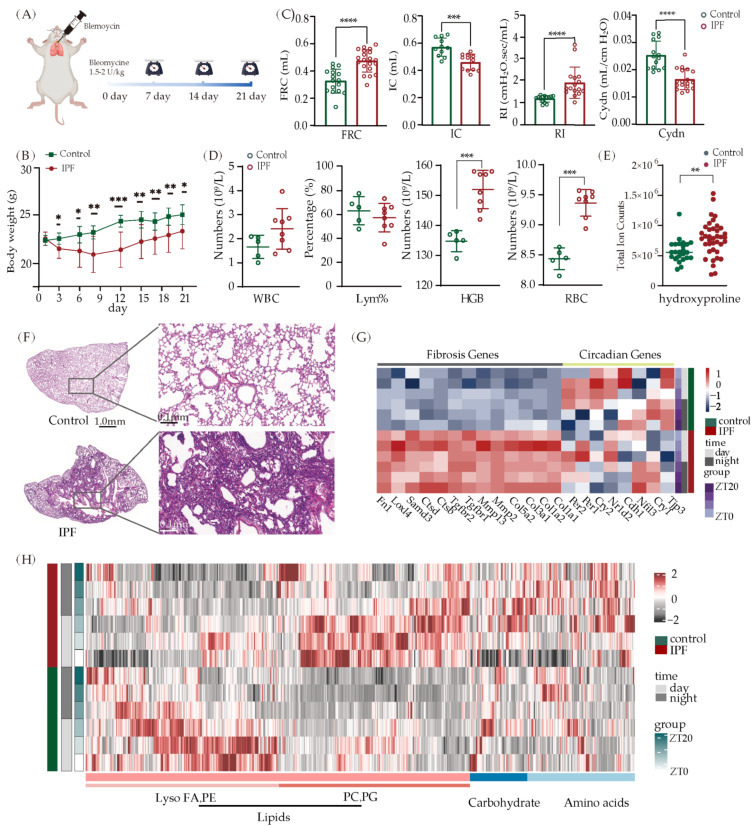
Lung rhythm oscillations in mice with IPF. (**A**) Schematic of IPF modeling. (**B**) Body weight changes during IPF progression in mice (IPF, *n* = 28; PBS, *n* = 6, * *p* < 0.05, ** *p* < 0.01, and *** *p* < 0.001). (**C**) Lung function in IPF mice. FRC: Functional Residual Capacity; IC: Inspiratory Capacity; RI: Resistance to Inhalation; Cydn: Dynamic Lung Compliance. (IPF, *n* = 20; PBS, *n* = 15, *** *p* < 0.001, **** *p* < 0.0001). (**D**) Changes of blood routine parameters in IPF mice. WBC: White Blood Cell Count; Lym%: Lymphocyte Ratio; HGB: Hemoglobin; RBC: Red Blood Cell Count. (*n* = 8, *** *p* < 0.001). (**E**) The detection of hydroxyproline in mice with IPF (*n* = 36, ** *p* < 0.01). (**F**) HE staining of lung tissue section from control mice and IPF mice. (**G**) Heatmap of genes related to fibrosis and circadian rhythm. (**H**) Temporal clustering curve of metabolites changes (*n* = 6).

By administering mice with bleomycin, we were able to construct an IPF model. Routine blood and pulmonary function tests were performed. Body weight was monitored every other day, and lung tissues were removed for pathological staining 21 days after modeling (Figure 3A). Compared to the control group, mice in the IPF group had significantly less body weight (Figure 3B). Lung function tests in the IPF group showed an increase in FRC and a decrease in IC (Figure 3C). Red blood cells (RBC) and hemoglobin levels were raised during the regular blood test, which may indicate hypoxia (Figure 3D). As the “gold standard” to estimate collagen content, hydroxyproline [42] is found with high concentrations in IPF (Figure 3E). Consistent with all these findings, histopathological examination of lung tissue sections stained with Hematoxylin and Eosin (H&E) revealed significant alterations in the structure of pulmonary parenchyma in the IPF group (Figure 3F). The sections displayed signs of lung tissue destruction, an increase in interstitial fiber proliferation, and the deposition of collagen proteins. These alterations reflect the pathological changes that are typical of pulmonary fibrosis.

To investigate whether circadian rhythm is changed in IPF, lungs from both the control and IPF models were collected for the identification of gene expression and the measurement of metabolites. We checked two types of gene expression patterns: circadian-related genes and fibrosis-related genes. The upregulation in fibrosis-related genes (Col3a1, Tgfbr1, Samd3, etc.) and disruption in circadian related genes (Per1, Per2, Cry1, Cry2, Nr1d2, etc) were observed (Figure 3G). In comparison to wild-type mice, the expression levels of Cry2 and Trp3 were found to be downregulated in individuals with IPF, while the expression levels of Per1 and Per2 showed a slight increase during the night time. The metabolic rhythm of IPF was also affected by the progression of fibrosis and the disturbance of circadian, resulting in significant alterations in lipid and amino acid profiles (Figure 3H). The rhythm of lysophosphatide and phosphatidylethanolamine (PE) displayed significant changes within the control group, while the phosphatidylcholine (PC) and phosphatidylglycerol (PG) rhythms exhibited noteworthy variations within the IPF group. The analysis of metabolite classification with rhythmic oscillations revealed that the number and types of metabolites varied to differing degrees. Similar to normal lung, the levels of lipids and amino acids also show rhythmic changes in IPF lung (Figure S3A). To examine how systemic metabolic rhythmic changes occurred in IPF, we analyzed metabolite changes at various reporting times throughout the day. The principal component analysis (PCA) analysis revealed separate clusters at ZT0 and ZT12 (Figure S3B). At ZT0 and ZT12, differential metabolite analysis revealed 97 and 46 altered metabolites, while 26 metabolites were altered at both time points (Figure S3B). Columnar accumulation diagram and volcano mapping of these metabolites suggested alterations in numerous amino acids and lipids (Figure S3C,D). These findings demonstrate that the circadian rhythm of the lungs was altered with the progression of fibrosis, at both the genetic level and the metabolic level.

### 3.4. Lipid Metabolism Disorders in IPF

**Figure 4:**
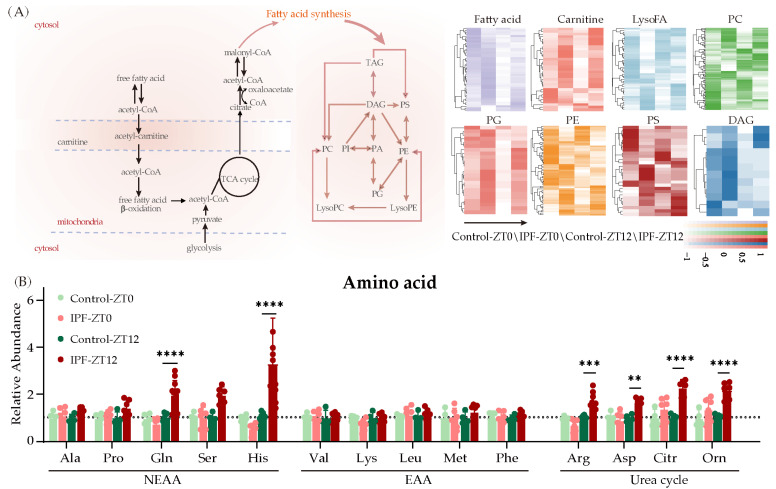
The changes of lipids and amino acids. (**A**) Lipid metabolism diagram and the heatmap of changed lipids. (**B**) Changes of amino acids in IPF mice (IPF, *n* = 8; control, *n* = 6, ** *p* < 0.01, and *** *p* < 0.001, **** *p* < 0.0001).

To gain a detailed understanding of the rhythmic oscillation of metabolites in IPF, we examined metabolite alterations classified using pathway separately. Lipid metabolism, which includes fatty acids, cholesterol, carnitine, etc., are essential components of cell membranes and play a crucial role in maintaining lung energy homeostasis. It has been reported that the dysregulation of lipid metabolism inhibits the regeneration of alveolar epithelial cells and promotes the transformation of fibroblasts into myofibroblasts, thereby contributing to the development of fibrosis [43,44]. Figure 4A exhibited the lipid changes in lung tissue (Figure 4A). There were significant differences in the concentrations of free fatty acid and carnitines between IPF and control groups at ZT0 and ZT12. Specifically, the level of free fatty acid reached its peak at ZT0, while carnitines had the maximum levels at ZT12. Moreover, a generally elevated level of these metabolites was observed in the presence of fibrosis. This suggests that the lungs with fibrosis either utilize less fatty acid oxidation for energy production or derive more lipid hydroxylation to maintain the fatty acid pool. Another observation was a disorder in the metabolism of PC, PE, and phosphatidylserine (PS). The majority of these lipids exhibit a distribution pattern opposite to that of IPF, while their circadian rhythm remains relatively unchanged. Diacylglycerol (DAG), an intermediate in the synthesis of PE and PS [43,45], appeared to dysregulate substantially at ZT0 but exhibited less pronounced changes at ZT12. These findings are supported by previous research from Yidan D. Zhao [46]. They analyzed a total of eight lung samples from individuals with IPF and eight lung samples from healthy donors, and their results were consistent with ours, demonstrating the dysregulation of lipid metabolism in IPF. However, when examining the energy production cycle, the tricarboxylic acid (TCA) cycle, we found that the levels of the majority of cycle intermediates were not significantly elevated between control and IPF; their metabolic rhythm was likewise stable (Figure S4A). Since amino acids (AAs) are another essential energy source and play a crucial role in cell signaling, we investigated the time dependencies of IPF’s effect on AAs metabolism [47]. Both essential and non-essential AAs were checked (Figure 4B), and the bar graph analysis revealed that their metabolism was strongly influenced by the time of day during IPF formation. The levels of glutamine, glutamate, serine, and tyrosine increased significantly at ZT12. Histidine levels decrease at ZT0, whereas rise at ZT12 (Figure S5A). Increased collagen deposition is a characteristic of fibrotic lung diseases. Glutamine, glutamate, and serine are all necessary for collagen synthesis [48]. It has been reported that histidine variation is associated with lung microbiota metabolism [49]. Histidine-rich glycoprotein (HRG) diets can promote the development of IPF [50]. Our findings highlight the complex dysregulation of lipid and amino acid metabolism along with circadian rhythm and fibrosis. Understanding the specific molecular mechanisms underlying these alterations could aid in the establishment of targeted treatments for pulmonary fibrosis.

### 3.5. The Urea Cycle Activity Increase in the Lungs of Mice with IPF

**Figure 5:**
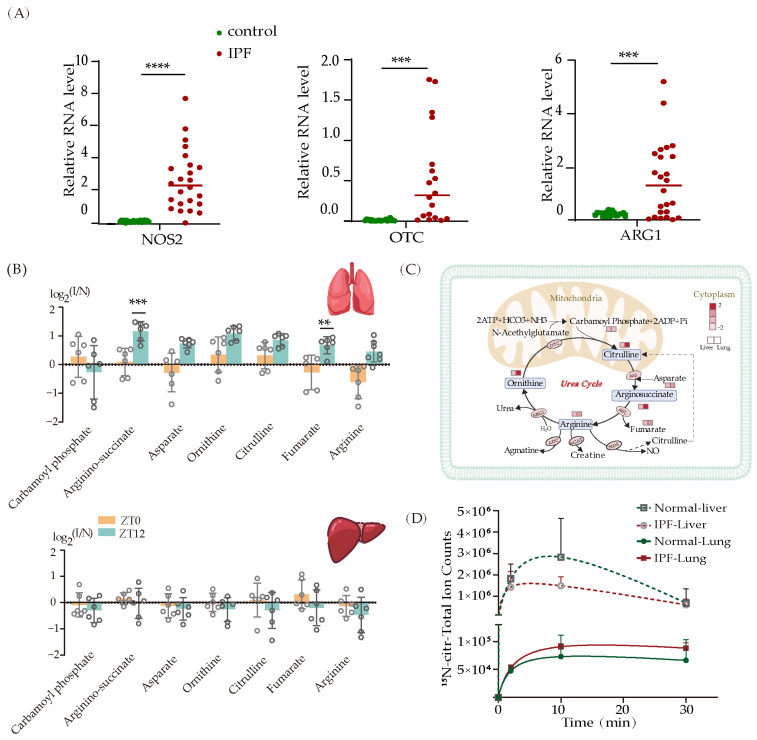
Urea cycle is upregulated in lung with IPF progression. (**A**) Changes in genes associated with the urea cycle (*n* = 20, *** *p* < 0.001, **** *p* < 0.0001). (**B**) Changes of metabolites in urea cycle at ZT0 and ZT12 in liver and lung of IPF mice (** *p* < 0.01, *** *p* < 0.001). (**C**) Schematic diagram of urea cycle metabolite changes. (**D**) The labeling curve of citrulline with ^15^N-ammonia injection.

In addition to the AAs listed above, we are also interested in the AAs implicated in the urea cycle, such as arginine, ornithine, citrulline, and aspartic acid. RNA sequencing of lung tissue demonstrated an increase in the expression of enzymes associated with the urea cycle, including OTC, ASS1, and ARG1 (Figure 5A and Figure S5B). Carbamoyl phosphate, ornithine, and citrulline were substantially upregulated at ZT0 in IPF lung tissue. Conversely, other urea cycle metabolites were significantly upregulated at ZT12 (Figure 5B). The urea cycle is a vital metabolic pathway that primarily takes place in the liver and converts toxic nitrogen compounds, such as ammonia, into non-toxic urea [51]. Interestingly, urea cycle metabolites in the liver were substantially downregulated at ZT12, the opposite of what was observed in the lung. To further explore the metabolic activity shift of the urea cycle in the lung and liver, we performed ^15^NH4Cl injection in both wild-type mice and mice with IPF. Blood, lung, and liver tissues were collected at 2 min, 10 min, and 30 min after injection, and the intensity of ^15^N-labeled citrulline was measured. As shown in Figure 5D, the intensity of ^15^N-labeled citrulline achieved a steady state within 30 min in both liver and lung. The higher labeling in the liver compared to the lung indicated a faster turnover of the urea cycle in the liver. However, upon examining the level of ^15^N-labeled citrulline between wild-type mice and mice with IPF, we observed an elevation in the intensity of ^15^N-labeled citrulline in the lung of IPF mice, while the intensity decreased in their livers. This demonstrates that the urea cycling flux in the lung is more rapid in IPF than in controls, which may compensate for the delayed urea cycling flux in the liver. Because the majority of ammonia originates from AA oxidation, specifically transamination of branch chain amino acids (Leu, Ile, and Val), Alanine, and deamination of glutamine, the rapid clearance of ammonia in the lungs suggests that more AAs may be oxidized for energy production to maintain TCA intermediate homeostasis.

## 4. Discussion

IPF is a severe chronic lung condition. Typically, its symptoms appear gradually and may not be noticed until the disease has become well-established [52]. The dysregulation of rhythmic gene expression suggests that rhythmic processes are involved in the development and progression of IPF [19,31,53]. Since metabolites are a crucial component of the cellular circadian clock, studies have increasingly focused on and shown that metabolic changes with circadian rhythms may play a significant role in the pathogenesis of IPF. Lipids are the primary building blocks of cell membranes and play a crucial role in cellular metabolism and growth regulation [54]. The Peroxisome Proliferated Activated Receptor (PPARα) serves as a major regulator of fatty acid oxidation. It has been suggested that the efficacy of lipid-lowering therapy with PPARα agonists is enhanced when administered during the daily peak of PPARα expression [55]. In our study, we observed changes in the levels of LysoFA and the intensities of PE, PC, and PG in the circadian metabolic rhythms of mice with IPF. Specifically, we noted a considerable increase in PC and PG during the daytime. Hence, the utilization of chronotherapy to modulate lipid metabolism presents a promising avenue for the treatment of IPF. Additionally, glycolysis and glucose metabolism are also altered in IPF, contributing to disease progression [56]. Xie et al. [57] reported that increased glycolysis stabilizes hypoxia-inducible factor 1α (HIF-1α), a factor required for myofibroblast differentiation. The stabilizing HIF-1α can also induce numerous physiological responses to compensate for the decreased tissue oxygen supply, including the production of erythropoietin (EPO), which can cause an increase in hemoglobin, may result in secondary erythrocytosis and pulmonary hypertension [58].

Energy is needed in large amounts by lung tissue to support various essential cellular functions, including reorganization of cytoskeletal components, gene transcription, protein translation, DNA replication, and repair [59]. Airway clearance via phagocytosis and ciliary movement, bronchial gland secretion, airway, and vascular constriction, and the production of pulmonary surfactants are all energy-intensive processes carried out by specialized lung cell populations. It has been discovered that fatty acids and lipids account for approximately 40% of the energy source in the lung, with carbohydrates and a trace quantity of amino acids coming the second and third. Under conditions of adequate oxygen, approximately 40% of the consumed glucose is ultimately converted into lactate [60,61], and the lactate generated can serve as an energy source for lung cells when the pulmonary circulation lacks sufficient nutrients [62]. It is still unclear how the lungs in IPF maintain energy balance. In this study, the rhythmic changes of lipids and amino acids suggested that energy production in the lungs may be reprogrammed from lipids or fatty acid oxidation to amino acid oxidation, which results in a buildup of lipids and a higher flux of the urea cycle to remove ammonia. By reprogramming the energy production pathway, this atlas will help to confirm the link between the development of IPF and circadian disruption, as well as the identification of potential IPF therapeutic targets.

## 5. Conclusions

One of the obstacles to achieving a comprehensive understanding of the rhythmic oscillation of metabolic changes in IPF is the lack of a complete metabolite database. In our study, isotopic labeling of yeast was employed in conjunction with the ESI-APCI ionization sources to establish a non-targeted metabolic database. This enables enhanced metabolite profiling by increasing precision and expanding the range of metabolites that could be identified. Following a series of metabolic analyses performed throughout a diurnal cycle and the administration of ^15^NH4Cl injections in both wild-type mice and mice with IPF, we have gained valuable insight into the metabolic characteristics of IPF, indicating that the modulation of the urea cycle, combined with chronotherapy, has the potential to slow down the onset and progression of IPF.

## 6. Limitation of the Study

This study primarily focused on water-soluble metabolites. The highly abundant and water-soluble lipids were also included. In order to determine which lipid-related pathway is dysfunctional in IPF, a deeper examination of all types of lipid changes is necessary. Our investigation was also restricted to a bulk analysis. The heterogeneity of lung tissue, with its diverse cell types and variable fibrosis pathology [52,63], should be taken into account in future studies. Although there is promise in the potential application of chronopharmacology and circadian biology in the management of IPF, additional consideration of other pulmonary fibrosis models that may represent IPF in humans, different genders, and ages are required for successful implementation.

## Data Availability

The data presented in this study are available in the article and Supplementary Materials.

## Supplementary Materials

The following supporting information can be downloaded at: https://www.mdpi.com/article/10.3390/metabo13101069/s1, Figure S1: Heat maps of meatbolites measured by different ion sources and extraction buffer, related to Figure 1; Figure S2: Venn diagram of all metabolites measured in different tissues, related to Figure 2; Figure S3: Classification of pulmonary metabolites in IPF mice, differential metabolites of ZT0,ZT12 and their classification, related to Figure 3; Figure S4: Analysis of differential metabolites of TCA in control and IPF mice lung, related to Figure 4; Figure S5: Changes of amino acids and RNA sequencing about ASS1 and GLUD1, related to Figure 5; Tables S1–S5 is related to Figure 1, Figure 2, Figure 3, Figure 4 and Figure 5; SI S1–S5 is related to Figures S1–S5.
