# Supplementary material for: Untargeted Metabolomics Reveals Alterations of Rhythmic Pulmonary Metabolism in IPF
Source: Metabolites. 2023 Oct 10;13(10):1069. doi: 10.3390/metabo13101069 (PMC10608701; doi:10.3390/metabo13101069)
Supplement: Supplementary file 1 [file metabolites-13-01069-s001.zip › SI.pdf]

metabolites

MDPI

Supplementary Materials

S1(A)

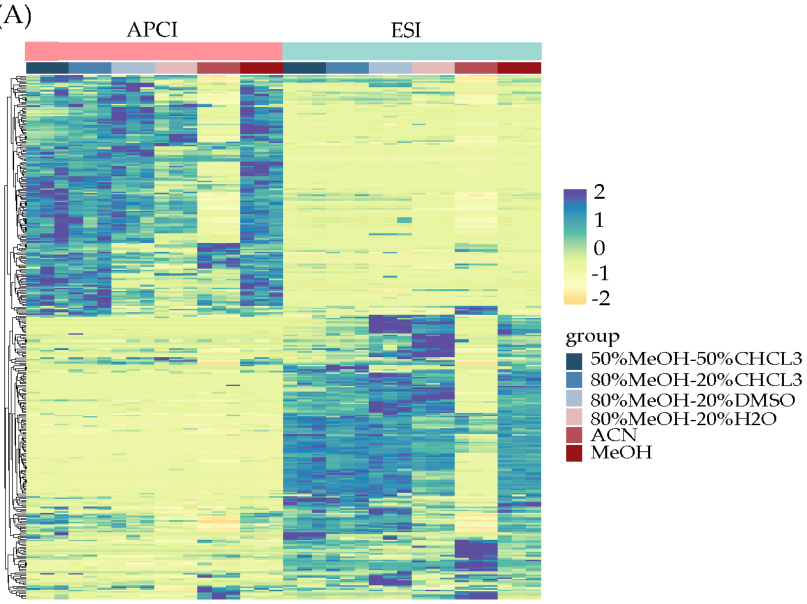

Figure S1. Heat maps of metabolites measured by different ion sources and extraction buffer.

S2(A)

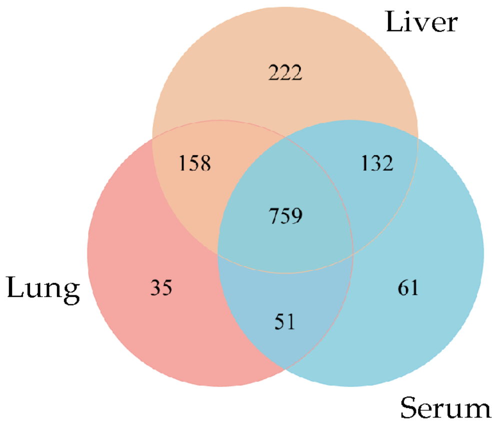

Figure S2. Venn diagram of all metabolites measured in different tissues

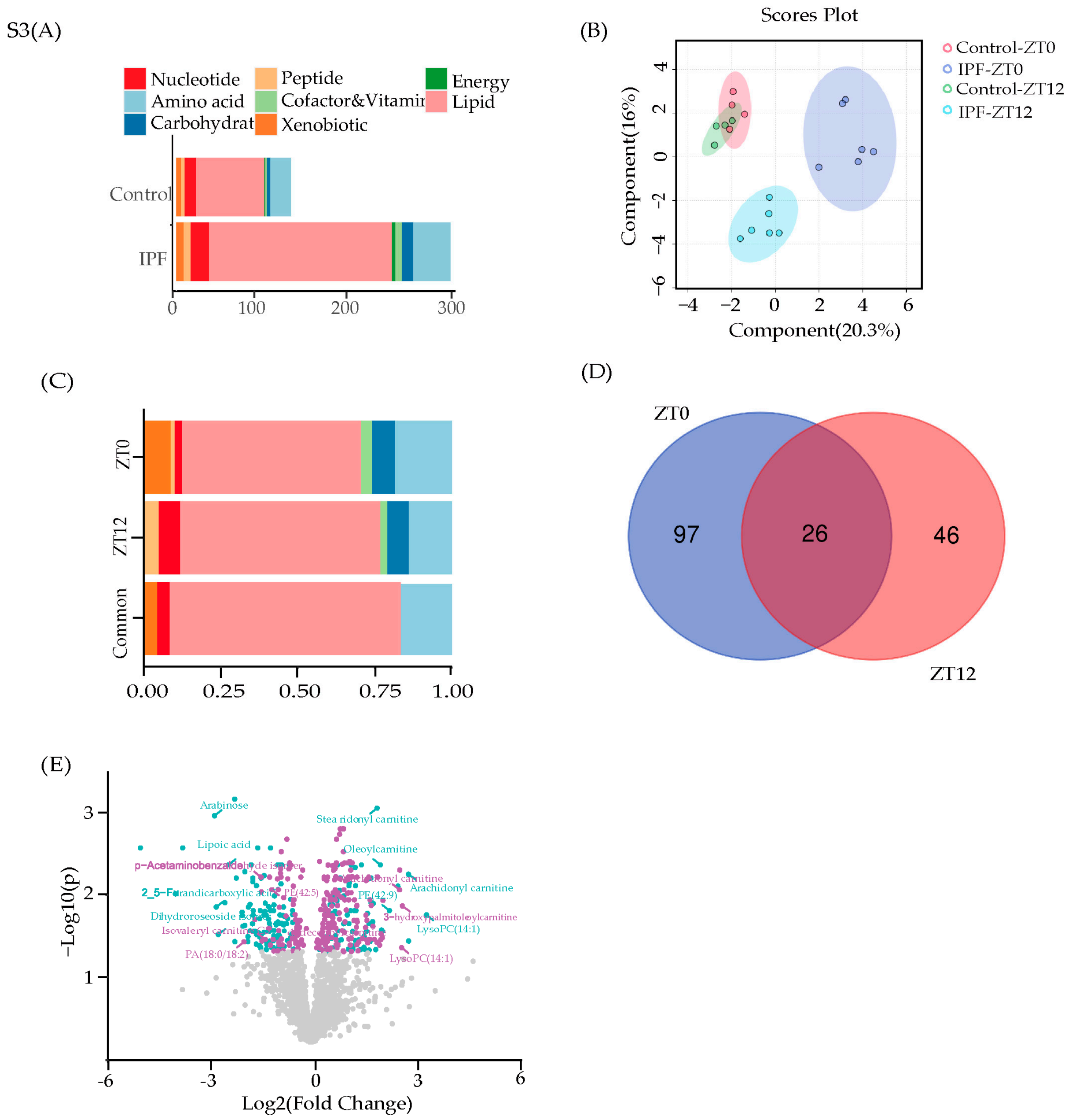

Figure S3. (A) Classification of metabolites with rhythmic oscillations for lung in control and IPF mice. (B) PCA analysis of circulatory metabolites in ZT0 and ZT12. (C) Classification of different metabolites at ZT0, ZT12 in control and IPF mice. (D) Number of metabolites with significant changes (control, *n*=4; IPF, *n*=6, *p* <0.05, log2 FC>1) when comparing lungs in control and IPF mice at ZT0 and ZT12. (E) Volcanic map of metabolites at ZT0, ZT12. Green represents ZT0 and purple represents ZT12.

S4(A)

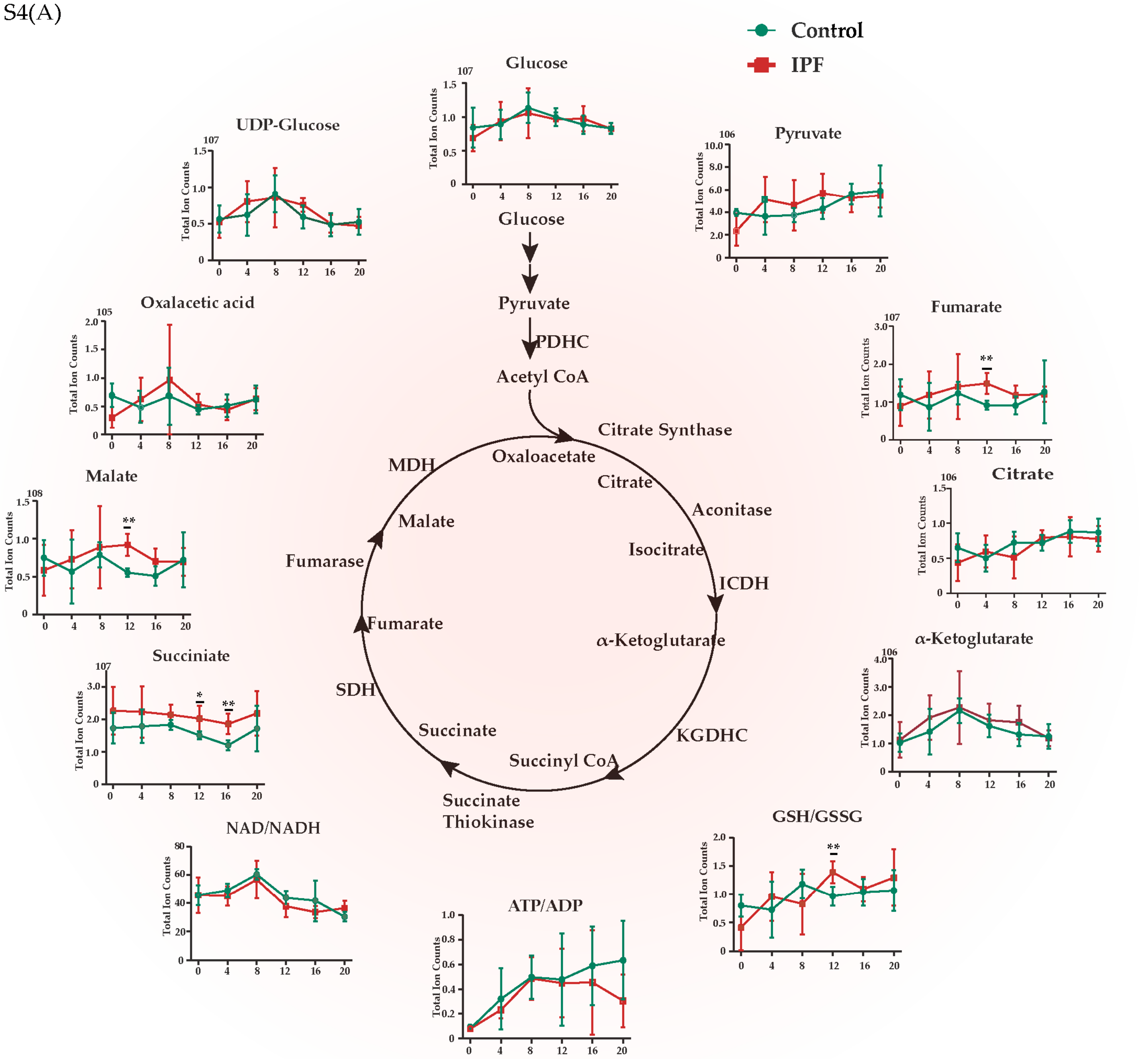

Figure S4. The rhythmic changes of metabolites at different time points of TCA cycle were observed in control and IPF mice (Control: *n*=4; IPF: *n*=6, *$p < 0.05$, **$p < 0.01$).

S5(A)

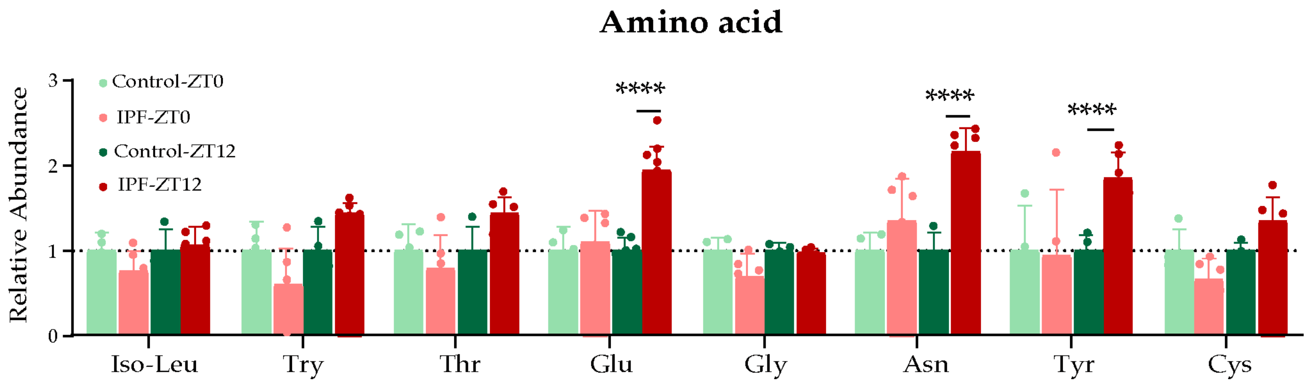

(B)

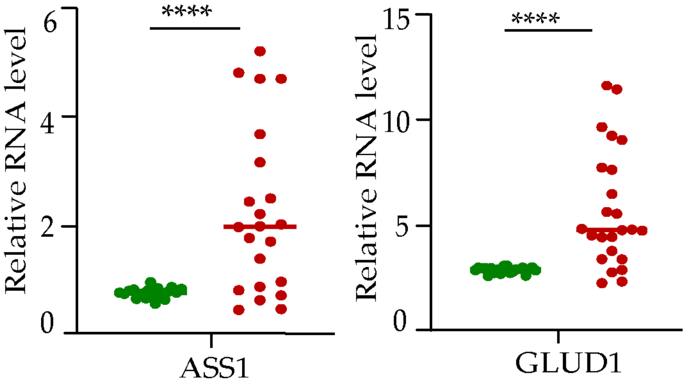

Figure S5. (A) Changes of amino acids in IPF mice .(IPF, n=8; control, n=6, ****$p < 0.0001$).(B) Changes in RNA sequencing about ASS1 and GLUD1.(n=20, ****$p < 0.0001$)
